# Outcome of lung oligometastatic patients treated with stereotactic body irradiation

**DOI:** 10.3389/fonc.2022.945189

**Published:** 2022-07-18

**Authors:** Guillaume Virbel, David G. Cox, Anne Olland, Pierre-Emmanuel Falcoz, Clara Le Fevre, Roland Schott, Delphine Antoni, Georges Noel

**Affiliations:** ^1^ Department of Radiation Oncology, Institut de Cancérologie Strasbourg Europe (ICANS), Strasbourg, France; ^2^ IRFAC – Statistic Department, INSERM U1113, Strasbourg, France; ^3^ Research and Development in Precision Medicine, Institut de Cancérologie Strasbourg Europe (ICANS), Strasbourg, France; ^4^ Department of Thoracic Surgery, Nouvel Hôpital Civil, Strasbourg University Hospital, Strasbourg, France; ^5^ Department of Medical Oncology, Institut de Cancérologie Strasbourg Europe (ICANS), Strasbourg, France

**Keywords:** lung, metastases, oligometastatic, stereotactic body radiotherapy, SBRT, SABR

## Abstract

**Purpose:**

The oligometastatic stage is an intermediate stage of cancer between the localized stage and polymetastatic stage. The prognosis of patients in this stage also appears to be intermediate. Lung stereotactic body radiotherapy is a possible tool for treating oligometastatic lung sites. The objective of our study was to evaluate the clinical outcomes in terms of local control, progression-free survival, overall survival, and toxicity of SBRT in oligometastatic patients with lung metastases from any solid primary tumor.

**Materials and methods:**

Clinical records of consecutive lung oligometastatic patients treated between January 2010 and December 2020 for lung SBRT at 60 Gy in 3- or 8-fraction schedules and a controlled primary tumor were retrospectively analyzed.

**Results:**

After a median follow-up of 20.3 months, local failure occurred for 14 lesions, 57 patients experienced lung progression, and 64 patients experienced disease progression. Overall survival rates at 1 and 2 years were 85.6 and 69.7%, respectively. Fifty-two patients experienced radiation pneumonitis, but only 2 patients were symptomatic and presented grade 2 late pneumonitis. No grade 3-4 toxicity was observed. ECOG 0 was the only prognostic factor for overall survival (HR = 3.5; 95% CI 3.2-3.8; p < 0.01).

**Conclusion:**

SBRT with a 60-Gy schedule in 8 fractions is an effective and well-tolerated treatment for patients with lung oligometastases from any solid primary tumor.

## Introduction

Treatment of metastatic disease is mainly based on systemic therapies. Since the description of oligometastatic disease in 1995 by Hellman and Weichselbaum, some patients with distant metastatic disease may benefit from a curative management strategy (1). Classic definition of oligometastases includes few organs affected by a limited number of metastases, but a consensus on the current definition has not been reached. Depending on the authors, the definitions have varied from 1 to 5 metastases in 1 to 3 sites (2–4). Some studies have shown an improvement in progression free survival (PFS) and overall survival (OS) by using strategies including focal treatments such as stereotactic radiotherapy when the primary tumor is controlled (2, 5).

The lung is the most frequent metastatic site for many cancers (6). Metastasectomy is the reference treatment with curative intent. However, some patients are not eligible for surgical treatment due to patient or tumor conditions (7, 8).

Lung stereotactic body radiotherapy (SBRT) is an effective and safe technique (9) that allows the use of a curative strategy despite operative contraindications or surgical refusal for patients with non-small cell lung cancer (NSCLC). Despite the lack of randomized trials, the reported results of retrospective series are comparable to those of surgical series (10, 11). As in NSCLC, SBRT is proposed to facilitate a curative strategy for patients with lung oligometastases. The combination of local and systemic treatments is not yet clearly defined for the management of these patients.

To clarify the treatment strategy, we report a series of oligometastatic patients treated in one comprehensive cancer center. The purposes of our retrospective study were to assess the local control (LC), PFS and OS rates of patients treated with lung SBRT for oligometastatic disease and to identify factors associated with each clinical outcome.

## Materials and methods

### Ethical approval

This study follows the mandatory French laws required by the CNIL (*Commission Nationale de l’informatique et des libertés*), was declared to this French institution by the MR004 form and was recorded in the HDH (Health Data Hub) and was approved by our Institutional Review Board.

### Inclusion criteria

Clinical records of consecutive patients with a controlled primary tumor treated by SBRT for lung metastases between January 2010 and October 2020 in a French comprehensive cancer center were collected. Lung metastases in adult patients with any solid primary tumor were included, with no restrictions in terms of systemic treatments for primary disease or metastases.

### Stereotactic body radiotherapy

All patients were simulated with a planning computed tomography (CT) scan (LightSpeed QX/I, GE Healthcare, US, Chicago) using a gating technique. CT scans with contrast injection were not mandatory. First, a 4D CT scan was performed to integrate tumor motion according to breathing movements. If the tumor displacement was greater than 1 cm in any direction, an inspiration breath-hold technique was performed (SDX^®^, Dyn’R Medical systems, France, Aix-en-Provence), or an abdominal compression plate (CIVCO Medical Solutions, US, Coralville, Iowa) was used when breathing was mainly abdominal. A planning PET/CT scan was performed for all patients.

An internal gross target volume (IGTV) was defined on the maximal intensity projection (MIP) sequence of the 4D CT scan. When PET/CT was performed, the biological target volume (BTV) was an aid to delineate the volume target. A 5-mm margin was added to the IGTV to define the internal clinical target volume (ICTV). When the breath-hold technique was used, the 4D scanner was not used. The gross tumor volume (GTV) was defined on the planning CT scan, and a 5-mm margin was directly added to the GTV to obtain the CTV. The isotropic margin between the ICTV or the CTV and the planning target volume (PTV) was 2 mm.

Treatments were delivered with dynamic conformal arcs using an iPlan System (Brainlab, Munich, DE) or volumetric modulated radiation therapy using an Eclipse System (Varian Medical Systems, Palo Alto, CA). A total dose of 60 Gy was prescribed to the isocenter, which was administered in 3 or 8 fractions of 20 or 7.5 Gy twice a week. A three-fraction protocol was preferentially chosen when lesion was more than 1 cm from chest wall and outside no fly zone. Acuros^®^ dose calculation algorithm used with the iPlan solution. MonteCarlo algorithm used with the Eclipse solution. Plans were developed such that the 80% isodose line encompassed the PTV, corresponding to a dose of 48 Gy. Quality criteria were analyzed, including the dose homogeneity and conformation index, according to the RTOG (12). Finally, all plans were judged as acceptable regarding the target coverage and sparing organs at risk. Treatments were delivered with a Novalis TX or a Novalis Truebeam STX (Varian medical system) linear accelerator with 6-MV photon energy.

### Follow-up and toxicities

After treatment, patients underwent thoracic re-evaluation with a CT scan, a PET/CT scan or both every 4 months for the first year, every 6 months in the second year and every year afterward.

Local failure was defined as new or progressive lesions arising in the radiation field on CT scans or metabolic imaging. New lung metastases outside the radiation field and local failure were defined as lung progression. Progressive disease was defined as new or progressive lesions at any site.

Acute and late toxicity was recorded prospectively using the Common Terminology Criteria for Adverse Events version 5.0 (13).

### Statistical analysis

Survival curves were calculated from the end of SBRT using the Kaplan–Meier method. The log-rank test was used to assess whether significant survival differences were present between the different groups. The BED_10 Gy_ values in the GTV, IGTV, CTV, ICV and PTV were calculated from the D_min_ delivered in the respective volumes, with the formula BED_10 Gy_ = nd*(1+d/α/β) where n = number of fractions, d: dose per fraction, α/β = 10 Gy. Prognostic factors were evaluated with respect to LC, lung progression-free survival (LPFS), PFS, and OS. All p values < 0.05 were considered statistically significant. Multivariate analysis was not performed because of the small number of events. These analyses were completed by R software.

## Results

### Patient characteristics

Corresponding to the inclusion criteria, 113 patients who underwent SBRT for lung metastases were enrolled. The sex ratio (M/F) was 1.6, and the median age at the time of irradiation was 68.8 years (min-max: 39-89). Ninety-eight (85%) patients had a 0 or 1 ECOG status. Eighteen (15.9%) patients were current smokers, 47 (41.6%) were former smokers, and 48 patients were not smokers or had no available data (**Table 1**). The median follow-up was 20.3 months.

**Table 1:** Patient, tumor, and treatment characteristics.

No of patients	113
Treated lesion number	141
Age: mean (Range) (years)	68.8 (39-89)
Sex (H/F)	
Male Female	70 (79.1%) 43 (20.9%)
Smoking history	
None Ex-smoker Current smoker Unknown	39 (34.5%) 47 (41.6%) 18 (15.9%) 9 (8%)
Primary disease	
Lung GI GU Head and Neck Resistant tumors Thyroid Breast Miscellaneous	34 (30.1%) 28 (24.8%) 18 (15.9%) 12 (10.6%) 8 (7.1%) 6 (5.3%) 5 (4.4%) 2 (1.8%)
Primitive disease histology	
Adenocarcinoma Squamous cell carcinoma Miscellaneous Neuroendocrine Sarcoma Melanoma Urothelial carcinoma:	55 (48.7%) 25 (22.1%) 19 (16.8%) 5 (4.4%) 5 (4.4%) 3 (2.7%) 1 (0.9%)
ECOG	
0 1 2 unknown	48 (42.5%) 48 (42.5%) 7 (6.2%) 10 (8.8%)
State of Oligometastatic Disease	
Synchronous metastases Metachronous metastases Oligoprogressive disease	3 (2.7%) 87 (77%) 23 (20.3%)
Diagnostic – SBRT time: mean (range) (month)	67.9 (2.1-388.5)
Other Treatments Before SBRT	
Prior RT (other than thoracic) Prior thoracic RT Prior thermoablation Prior thermoablation of treated lesion Prior lung metastasectomy Prior lung cancer surgery (primitive) Prior systemic therapy regimens for metastatic disease Prior systemic therapy regimens for primitive disease Prior chemotherapy Prior hormonotherapy Prior target therapy Prior immunotherapy	45 (39.8%) 33 (29.2%) 8 (7%) 5 21 (18.6%) 18 (15.9%) 56 (49.6%) 56 (49.6%) 72 (63.7%) 5 (4.4%) 22 (19.5%) 5 (4.4%)
Only lung involved at Time of SBRT (113 patients)	
Yes No	93 (82.3%) 20 (17.7%)
Number of treated lesions per patient	
1 2 3 4	88 (77.9%) 23 (20.3%) 1 (0.9%) 1 (0.9%)
Lesion’s location	
No-fly zone < 1 cm to thoracic wall	12 (8.5%) 49 (34.8%)
breath hold technic	40 (28.4%)
Diameter: mean (Range) (cm)	1.59 (0.46-5.55)
GTV: median (range) (cm3)	1.8 (0.1-45.0)
IGTV: median (range) (cm3)	2.6 (0.6-23.7)
CTV: median (range) (cm3)	9.4 (2.35-91.8)
ICTV: median (range) (cm3)	12.6 (4.8-59.5)
PTV: median (range) (cm3)	17.4 (5.4-123.1)
SBRT dose prescription	
3 x 20 Gy 8 x 7.5 Gy	11 (7.8%) 130 (92.2%)
Treatment technique	
Dynamic Arctherapy VMAT	107 (75.9%) 34 (24.1%)

### Tumor and treatment characteristics

Patients underwent lung SBRT for 141 lung metastases. The two most frequent primary sites were the lungs and the gastrointestinal tract, which were affected in 34 (30.1%) and 28 patients (24.8%), respectively. More than two-thirds of the tumors were adenocarcinoma (48.7%) or squamous cell carcinoma (22.1%). The median time interval between primary tumor diagnosis and lung SBRT was 42 months (2.1-388.5). Before SBRT, forty-five patients (39.8%) had received radiotherapy for another tumor location, and 33 (29.2%) had received thoracic radiotherapy for their primary tumors. Thermoablation before SBRT for the same lesion or for other lung metastases was performed in five and eight patients, respectively. Surgery for primary lung cancer or metastases was performed in 18 (15.9%) and 21 patients (18.6%), respectively. Most patients had received systemic therapy prior to SBRT. Details of these treatments are reported in **Table 1**.

At the time of treatment, 87 (77%) patients presented metachronous oligometastatic disease, 23 (20.3%) developed oligoprogressive disease and 3 had synchronous oligometastatic disease. The lung was the only metastatic site in 93 patients (82.3%). One metastasis was irradiated in 88 patients (77.9%), two in 23 patients (20.3%), and three or four in one patient (0.9%) each time. Twelve (8.5%) metastases were in the no-fly zone, and 49 (34.8%) were at 1 cm or less in the chest wall (14).

The median lesion diameter was 1.59 cm (0.46-5.55). The median PTV volume was 17.4 cm^3^ (5.4-123.1). An IGTV was defined for 101 (71.6%) lesions. A GTV was delineated for the 40 (28.4%) remaining lesions. The prescribing doses were 60 Gy in 3 fractions for 11 (7.8%) lesions and 60 Gy in 8 fractions for 130 (92.2%) lesions. According to the dose into the CTV, the equivalent dose with an α/β ratio at 10 Gy and the biologically effective dose (BED_10 Gy_) were 124.8 Gy and 76.8 Gy, for the schedules delivering 3 and 8 fractions respectively; also, median conformity and homogeneity indices were 0.997 and 0.214, respectively. For 3 fractions, the mean minimum BED_10 Gy_ values in the GTV, IGTV, CTV, ICTV and PTV were 150.6 Gy_BED_ (95 percent confident interval (95CI): 125,8 – 175,4), 150,5 Gy_BED_ (95CI: 148,2 – 152,8), 141,9 Gy_BED_, 137,6 Gy_BED_ (95CI: 137,1 – 138,3) and 126,3 Gy_BED_ (95CI: 122,1 – 130,4) respectively; for 8 fractions, these values were 91,8 Gy_BED_ (95CI: 90,0 – 93,6), 92,7 Gy_BED_ (88,4 – 97,0), 85,1 Gy_BED_ (95CI: 83,7 – 86,5), 87,4 Gy_BED_ (95CI: 86,4 – 88, 5) and 75,02 Gy_BED_ (95CI: 74,1 – 76,0) respectively.

### Local control

**Figure 1:**
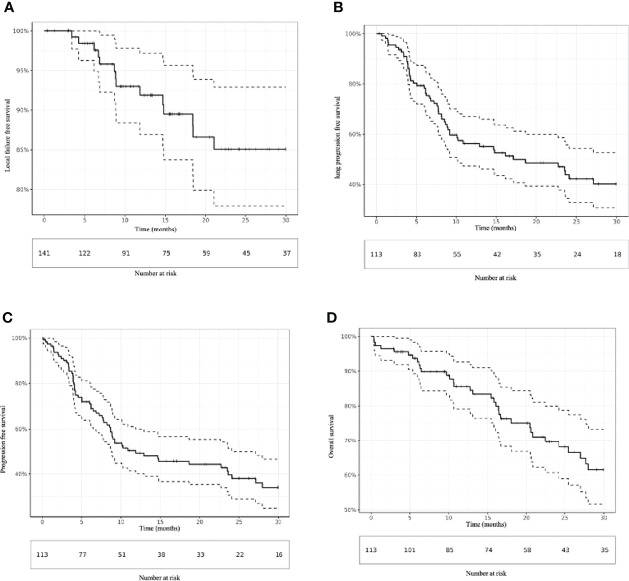
Kaplan–Meier estimation of **(A)** local failure-free survival, **(B)** lung progression-free survival, **(C)** progression-free survival and **(D)** overall survival.

Local failure occurred for 14 lesions (9.9%), with a median time of 8.9 months (range, 3.4-21.1 months). The 6-, 12-, and 24-month LC rates were 98.4% (95% CI [confidence interval] 96.3-100), 91.9% (95% CI 86.9%-97.2%), and 85.1% (95% CI 77.9%-92.9%), respectively (**Figure 1**). According to univariate analysis, an ECOG greater than 0 (HR = 11.1 95% CI 10.3-11.9; p = 0.016) were associated with an increase in LF. No significant association between metastasis size (p = 0.82), primary colorectal disease (p = 0.32), pre-SBRT systemic treatment (p = 0.2) and BED_10 Gy_ (p = 0.46) was found. LC by age was not analyzed because of the absence of events for the oldest patients (**Table 2**).

**Table 2:** Univariable analysis of prognostic factors.

Local control				
Variable	Level	N (%)	Hazard Ratio (95%CI)	p Value
ECOG	0	1 (1.5%)	1.0 (ref)	
	> 0	7 (11.3%)	11.1 (10.3-111.9)	p = 0.016
**Lung progression-free survival**				
Variable	Level	N (%)	Hazard Ratio (95%CI)	p Value
Number of lung metastases	Single lung metastasis	38 (42.2%)	1.0 (ref)	
	Multiple lung metastases	19 (65.5%)	3.9 (3.2-4.6)	p = 0.017
Oligoprogressive disease	Other	39 (43.3%)	1.0 (ref)
	Oligoprogressive disease	18 (78.3%)	11.9 (8.4-16.8)	p < 0.001
Oligometastatic state	Other	20 (79.9%)	1.0 (ref)
	Metachronous oligometastatic state	37 (42.5%)	0.10 (0.07-0.13)	p < 0.001
**Progression-free survival**				
Variable	Level	N (%)	Hazard Ratio (95%CI)	p Value
Number of lung metastases	Single lung metastase	43 (51.2%)	1.0 (ref)
	Multiple lung metastases	21 (72.4%)	3.9 (3.2-4.7)	p < 0.01
Oligoprogressive disease	Other	45 (50.0%)	1.0 (ref)
	Oligoprogressive disease	19 (82.6%)	10.5 (7.0-15.6)	p < 0.001
Oligometastatic state	Other	21 (80.8%)	1.0 (ref)
	Metachronous oligometastatic state	43 (49.4%)	0.11 (0.08-0.16)	p < 0.001
**Overall survival**				
Variable	Level	N (%)	Hazard Ratio (95%CI)	p Value
ECOG	0	12 (25.0%)	1.0 (ref)
	> 0	28 (50.9%)	3.5 (3.2-3.8)	p < 0.01
**Radiation pneumonitis**				
Variable	Level	N (%)	Hazard Ratio (95%CI)	p Value
Age	< 65 years	13 (28.3%)	1.0 (ref)
	> 65 years	52 (64.2%)	3.6 (3.2-4.0)	p < 0.001
V10Gy	< 351 cc	21 (42.0%)	1.0 (ref)
	> 351 cc	30 (66.7%)	4.7 (4.1-5.4)	p = 0.022
V35Gy	< 32.9 cc	18 (36.0%)	1.0 (ref)
	> 32.9 cc	33 (71.7%)	5.1 (4.4-5.9)	p < 0.01

### Lung progression-free survival and progression-free survival

In total, 57 (50.4%) patients experienced lung progression, with a median of 7.2 months (range, 0.7-54.8 months). The 6-, 12-, and 24-month LPFS rates were 79.3% (95% CI 72.0-87.4), 56.3% (47.3-67.0), and 43.8% (34.4-55.7), respectively (**Figure 1**). On univariate analysis, multiple lung metastases (HR = 3.9; 95% CI 3.2-4.6; p = 0.017) and oligoprogressive disease (HR = 11.9; 95% CI 8.4-16.8; p < 0.001) were associated with the worst LPFS. In contrast, a metachronous oligometastatic state (HR = 0.10 95%CI 0.07-0.13; p<0.001) was associated with better LPFS.

Sixty-four (56.6%) patients experienced progressive disease, with a median of 6.2 months (range, 0.1-42.2 months). The 6-, 12-, and 24-month PFS rates were 72.0% (95% CI 64.0-81.0), 49.1% (40.2-60.0), and 39.6% (30.6-51.3), respectively (**Figure 1**). Similar to LPFS, univariate analysis showed that multiple lung metastases (HR = 3.9; 95% CI 3.2-4.7; p < 0.01) and oligoprogressive disease (HR = 10.5 95%CI 7.0-15.6; p < 0.001) were associated with worse PFS. A metachronous oligometastatic state (0.11 95% CI 0.08-0.16; p < 0.001) was associated with better LPFS (**Table 2**).

### Overall survival

Forty-four patients (38.9%) died within a median time of 16.6 months (range, 0.3-66.6). The 6, 12-, and 24-month OS rates were 92.7% (95% CI: 88.0-97.7), 85.6% (79.1%-92.7%), and 69.7% (60.7%-80.0%), respectively (**Figure 1**). According to the univariate analysis, an ECOG greater than 0 was the only prognostic factor for OS. The 6, 12-, and 24-month OS rates for patients with ECOG 0 were 97.9% (95% CI: 94.0-100.0), 92.6% (84.9%-100.0%) and 83.6% (72.3%-96.7%), respectively compared to 87.1% (95% CI: 78.6-96.5), 77.2% (66.6%-89.5%) and 59.9% (47.1%-76.3%) respectively for patients with ECOG >0 (HR = 3.5; 95% CI 3.2-3.8; p < 0.01) (**Table 2**).

### Toxicities

No severe acute or late side effects (grade >2) were observed. Fifty-two (46.0%) patients experienced radiation pneumonitis (RP), but only two patients were symptomatic and presented grade 2 late pneumonitis. Grade 1 acute parietal pain was observed in three patients, but no rib fracture was recorded. Grade 1 esophagitis occurred in four patients (3.5%).

Age > 65 years (HR = 3.5; 95% CI 3.2-4.0; p < 0.001), a V_10Gy_ > 351 mL (HR = 4.7; 95% CI 4.1-5.4; p = 0.022) and a V_35Gy_ > 32.9 mL (HR = 5.1; 95% CI 4.4-5.9; p < 0.01) were associated with a higher probability of radiologic RP. In contrast, the use of a 3-fraction schedule (p = 0.79) and active smoking (p = 0.49) did not increase the risk of RP (**Table 2**).

### Salvage therapy

Among the 12 patients with local failure, three patients received local salvage therapy with new SBRT or radiofrequency ablation. Six patients received a systemic therapy. Twenty-five (22%) patients received focal treatment for oligoprogression, and 17 (15.0%) patients received new lung SBRT. One patient received radiofrequency ablation. Thirty-nine patients received systemic treatment for distant progression.

## Discussion

We reported the largest series of patients treated for lung metastases with fractionated homogeneous ablative dose (15). The main objective of SBRT was definitive LC of the irradiated lesion. The LC rate in this study was 91% at one year. A systematic PRISMA review reported LC rates from 18 published studies between 2016 and 2021, which ranged from 31% to 93%. Our result is in the higher range of those reported in the most recent series (15). These results are also comparable to those in surgical series reporting complete resection in 88% of cases. The 5-year survival rates after complete and partial metastasectomy were 36% and 13%, respectively (6). These results encourage the use of focal therapies to achieve LC of oligometastases with an OS improvement intent.

Some lung SBRT studies found that lesion size was associated with LC. Osti et al. and Jung et al. found threshold values of 1.8 cm and 2.5 cm, respectively (16, 17). No statistical relationship with lesion size was found in this study. The homogeneity of the size of the different irradiated lesions may explain this result.

A BED_10 Gy >_100 Gy, into the GTV or PTV with a small margin, was also found to be a factor for a better local prognosis, as in NSCLC (18–21). However, one must be cautious with BED_10 Gy_ because the method of reporting or calculating the doses was not systematic (18, 20). In our series, the most comparable BED_10 Gy_ with those used by previous publication, was the BED_10 Gy_ delivered into the GTV because of the used margin in our protocol. Patients treated with 3 fractions received a largely high BED_10 Gy_, larger than 100 Gy, and those treated with 8 fractions had a very close 100 Gy BED_10 Gy_, but with a small confidence interval (CI). These both results (i.e. high BED_10 Gy_ and small CI) explain likely why this BED_10 Gy_ was not a prognosticator.

Primary CRC was not an LC prognostic factor, as reported in some recent studies. In a retrospective series of 129 patients including 41 CRC lung metastases, the 3-year LC rates in patients with lung metastases from CRC and non-CRC were 64.8% and 86.3% (p < 0.01), respectively (17). Helou et al. reported cumulative incidence rates of local failure of 23.6% and 8.3% for CRC and non-CRC metastases, respectively (p < 0.001). Doses of 48 to 52 Gy in 4 or 5 fractions were delivered (20). The dose schedule used in the current study (60 Gy in 3 or 8 fractions) could eliminate the potential difference in LC between CRC and non-CRC metastases.

The most used radiation schedule in the current series was 60 Gy in 8 fractions. This schedule corresponds to a BED_10 Gy_ of 76.8 Gy, which is one of the smallest doses described in the literature. However, the 2-year LC rate of 84.2% was comparable to those obtained with higher doses. Wang et al. reported a 2-year LC rate of 90.2% using a mostly BED_10 Gy_ above 100 Gy. However, Wang et al. obtained the PTV by adding a 3-mm margin directly to the GTV. No CTV was noted, which complicates comparisons of the studies and may explain the lack of difference in results despite the BED_10 Gy_ difference. Notably, in the current series, the GTV and IGTV median minimum BED_10 Gy_ were 151.2 Gy and 155.4 Gy for 3 fractions and 94.6 Gy and 95.4 Gy for 8 fractions, respectively. No difference in terms of LC was found between the two groups. The absence of a CTV margin provided a similar LC but required the use of larger doses (18). A specified CTV and a required dose to cover it could improve the homogeneity of the dose distribution and consequently increase the control of the disseminated or migrating cells around the GTV.

In the present study, SBRT was well tolerated. Only two symptomatic RP cases were identified. Menoux et al. reported a series of 90 patients with NSCLC treated with lung SBRT. They found a rate of 26% for symptomatic RP with the same schedules. The difference with the current cohort can be explained by the differences in lesion size and in the irradiated healthy lung volume. In this series, the average size of the metastases was 1.59 cm. Menoux et al. reported a majority of cancers > 2 cm (22). Furthermore, for primary tumors, Menoux et al. reported a GTV-CTV margin of 5, 6 or 8 mm according to pathology, although we mainly used a 5-mm margin. Consequently, this low rate of radiation pneumonitis can be explained by the volumes of the targeted volumes, as some authors have already shown (11).

In the current study, fractionation was not predictive of RP risk (p = 0.79), which is consistent with the results of a phase 2 randomized study comparing single-fraction SBRT schedules with 4-fraction schedules for lung oligometastases. The results of this trial did not show any difference in grade 3 or higher toxicity between the two groups (23).

Oligometastatic disease represents a major challenge because it affects patients potentially eligible for curative treatment. The 13-month improvement in overall survival in the SABR COMET trial has prompted the use of ablative therapy for these patients (2). The challenge is to select patients whose disease will not progress to an incurable polymetastatic stage. The type of oligometastatic disease is an important element for this selection. The current series showed that patients with metachronous oligometastatic cancer seem to have a better prognosis. In contrast, those with oligoprogressive oligometastatic disease have poorer outcomes. This finding has been found in several other studies involving SBRT of lung metastases (19, 24, 25). In a retrospective study including 30 patients who underwent lung metastasectomy and 21 patients who received lung SBRT, Lee et al. found that synchronous metastasis was associated with poor OS (p = 0.026) (24). In a retrospective study of lung SBRT metastases from CRC, the oligoprogression group had a worse one-year regional metastasis rate than the oligometastasis group (79.5% vs. 25.1%, p = 0.001) (25). In a third study, 206 patients underwent SBRT for lung oligometastases, and the presence of synchronous metastases was an independent factor associated with poor LC (HR 2.21, 95% CI 1.22–4.00, p = 0.009) (19). These results can be explained by the different metastatic potentials of these oligometastatic diseases. An early metastatic disease has greater potential for dissemination than an initially localized disease that has disseminated secondarily (26). In a study comparing ultrahigh single-dose radiation therapy (SDRT) (24 Gy in one fraction) and fractionated SBRT (27 Gy in 3 fractions), Zelefsky et al. theorized that oligometastatic syndrome was a biologically dynamic process associated with the evolution of a propensity for distant metastatic spread along a linear continuum that progressively shifts the metastatic equilibrium toward polymetastatic conversion (27).

Intensifying treatment of these patient subgroups appeared to be required. In the SABR COMET study, all patients received standard systemic therapy (2). In a specific study on oligometastatic NSCLC, patients remaining oligometastatic after a first line of systemic therapy were randomized between maintenance therapy and local treatment of the entire lesion +/- maintenance therapy. Twenty percent of the test group received systemic therapy complementary to local therapy (28). While local treatment has resulted in an OS gain, its place with systemic treatment remains disputable. Immunotherapy could be positively combined with SBRT. Indeed, high doses of radiotherapy generate systemic immune changes that could stimulate the immunologic system of the patient. This immune reaction could be improved by combination with immunotherapy (29, 30). However, this combination needs more prospective trials to be used in practice (31).

One strategy to improve the prognosis of patients with oligometastases is to better select them. PET/CT would allow reclassification of patients with too many metastases. Rieber et al. found better OS if PET/CT was performed before SBRT of 700 lung metastases (32). The use of microRNAs could also help to distinguish patients who are progressing to incurable metastatic disease (33) but remains in development.

Node status was a prognostic factor conditioning survival in patients treated with local therapy for oligometastatic disease in some retrospective studies. This factor is notably found for NSCLC (34, 35). Because of the variability of the primary cancers included in this study, lymph node status was not collected.

ECOG greater than 0 was the only prognostic factor for overall survival. Although intuitively the better the ECOG, the better the chance of staying alive for a long time whatever the delivered treatment. This result should be used to highly recommend this SBRT for patient unsuitable or refusing surgery when their ECOG status is 0. However, considering the high response rates in the complete series, SBRT may not be reserved only for the patients with ECOG 0.

The current study has some limitations. Mainly, the heterogeneity of primary cancers may slow the development of the technique, but this group of patients treated with local therapy may be comparable to those in basket trials where patients with several kinds of pathology can be included (15). Furthermore, this heterogeneity is inherent to this kind of large series studying not the primitive tumors but metastases (17–21); the series studying only one subtype of metastasis collected a smaller number of patients (16, 25, 36–39). A larger cohort could offer an adapted personalized strategy according to the cancer subtypes.

To evaluate the relevance of this treatment in various cancer sites, several trials involving other cancer sites are ongoing. STEREOSARC (NCT02089100) is a prospective, multicenter, randomized, open-label phase II study comparing immunomodulation with atezolizumab plus SBRT versus SBRT alone in patients with oligometastatic sarcoma. The systemic immune effect of SBRT and the action of anti-PD-L1 synergistically increase systemic antigen release and thus enhance antitumor action (40). STEREO-SEIN (NCT02089100) is a multicenter phase III trial comparing SBRT plus systemic therapy to systemic therapy alone in patients with metastatic breast cancer as first-line therapy (41). SABR-COMET-3 and SABR-COMET-10 are randomized phase III studies including patients with 1-3 and 4-10 metastases, respectively, and comparing SBRT plus standard therapy versus standard therapy alone (42, 43). However, similar studies have yet to be performed for patients with lung oligometastases.

## Conclusion

SBRT with a 60-Gy schedule in 8 fractions is an effective and well-tolerated treatment for patients with pulmonary oligometastases from any primitive tumor. With a median follow-up of 20.3 months, the 2-year LC rate was 84.2%, and no acute or late grade 2 complications were observed. Hence, SBRT is an ablative treatment that should be integrated into therapeutic management.

## Data availability statement

The raw data supporting the conclusions of this article will be made available by the authors, without undue reservation.

## Ethics statement

The studies involving human participants were reviewed and approved by Health data hub with the number F20210209131317. The patients/participants provided their written informed consent to participate in this study.

## Author contributions

GV: Conceptualization, Methodology, Formal analysis, Investigation, Writing - Original Draft; DC: Formal analysis; AO: Writing - Review and Editing; P-EF: Writing - Review and Editing; CL: Writing - Review and Editing; RS: Writing - Review and Editing; DA: Writing - Review and Editing; GN: Conceptualization, Methodology, Writing - Review and Editing. All authors contributed to the article and approved the submitted version.

## Conflict of interest

The authors declare that the research was conducted in the absence of any commercial or financial relationships that could be construed as a potential conflict of interest.

## Publisher’s note

All claims expressed in this article are solely those of the authors and do not necessarily represent those of their affiliated organizations, or those of the publisher, the editors and the reviewers. Any product that may be evaluated in this article, or claim that may be made by its manufacturer, is not guaranteed or endorsed by the publisher.
